# Deciphering the Mechanism of Gilteritinib Overcoming Lorlatinib Resistance to the Double Mutant I1171N/F1174I in Anaplastic Lymphoma Kinase

**DOI:** 10.3389/fcell.2021.808864

**Published:** 2021-12-23

**Authors:** Shuai Liang, Qing Wang, Xuesen Qi, Yudi Liu, Guozhen Li, Shaoyong Lu, Linkai Mou, Xiangyu Chen

**Affiliations:** ^1^ Department of Urology, Affiliated Hospital of Weifang Medical University, Weifang Medical University, Weifang, China; ^2^ Oncology Department, Xinhua Hospital Affiliated to Shanghai Jiao Tong University, School of Medicine, Shanghai, China; ^3^ Medicinal Chemistry and Bioinformatics Center, Shanghai Jiao Tong University, School of Medicine, Shanghai, China; ^4^ School of Medical Laboratory, Weifang Medical University, Weifang, China

**Keywords:** anaplastic lymphoma kinase, non-small cell lung cancer, targeted therapy, drug resistance, molecular dynamics simulations

## Abstract

Anaplastic lymphoma kinase (ALK) is validated as a therapeutic molecular target in multiple malignancies, such as non-small cell lung cancer (NSCLC). However, the feasibility of targeted therapies exerted by ALK inhibitors is inevitably hindered owing to drug resistance. The emergence of clinically acquired drug mutations has become a major challenge to targeted therapies and personalized medicines. Thus, elucidating the mechanism of resistance to ALK inhibitors is helpful for providing new therapeutic strategies for the design of next-generation drug. Here, we used molecular docking and multiple molecular dynamics simulations combined with correlated and energetical analyses to explore the mechanism of how gilteritinib overcomes lorlatinib resistance to the double mutant ALK I1171N/F1174I. We found that the conformational dynamics of the ALK kinase domain was reduced by the double mutations I1171N/F1174I. Moreover, energetical and structural analyses implied that the double mutations largely disturbed the conserved hydrogen bonding interactions from the hinge residues Glu1197 and Met1199 in the lorlatinib-bound state, whereas they had no discernible adverse impact on the binding affinity and stability of gilteritinib-bound state. These discrepancies created the capacity of the double mutant ALK I1171N/F1174I to confer drug resistance to lorlatinib. Our result anticipates to provide a mechanistic insight into the mechanism of drug resistance induced by ALK I1171N/F1174I that are resistant to lorlatinib treatment in NSCLC.

## Introduction

As a transmembrane receptor tyrosine kinase, anaplastic lymphoma kinase (ALK) is a member of the insulin receptor tyrosine kinase superfamily. ALK is mainly expressed in adult brain tissue and plays an essential role in the function of central and peripheral nervous systems (Golding et al., 2018). Accumulating evidence indicates that gene amplification in the ALK domain or the acquisition of activating point mutations have been found in neuroblastoma, anaplastic large cell non-Hodgkin’s lymphoma, diffuse large B-cell lymphoma, and non-small-cell lung cancer (NSCLC) (Golding et al., 2018; Indini et al., 2020). Specially, rearrangements in the ALK are responsible for ∼3–5% of advanced NSCLC oncogenic driver mutations. Thus, ALK has been considered as an important therapeutic target for the treatment of NSCLC and various blood tumors harboring an ALK fusion (Roskoski, 2017; Kong et al., 2019; Yang and Gong, 2019; Chhikara et al., 2020; Chen et al., 2020b).

In the past decade, mammoth efforts have been paid to discover and develop ALK inhibitors. For example, crizotinib that bound to the ATP-binding site of ALK kinase domain was the first ALK inhibitor approved by the U.S. FDA in 2011 in the first-line treatment of ALK-positive NSCLC patients (Cui et al., 2011). Unfortunately, the clinically acquired mutations of ALK such as the L1196M gatekeeper mutation, I1171T, F1174C, G1202R, S1206Y, and G1269A mutations render crizotinib treatment ineffective (Carpenter and Mossé, 2012). Such drug-resistant mutations have witnessed a recent upsurge fueled by the growing interest in the development of second-generation ALK inhibitors such as ceritinib, alectinib, and brigatinib for treatment of advanced (metastatic), ALK-positive NSCLC patients who had no response with crizotinib treatment (Roskoski, 2017; Roskoski, 2021). Recently, the third-generation ALK inhibitor lorlatinib has been received an accelerated approval by the U.S. FDA for patients with ALK-positive NSCLC whose metastatic disease were ineffective in response to targeted therapies such as crizotinib, ceritinib, alectinib, and brigatinib (Johnson et al., 2014). Clinical trails showed that lorlatinib had marked therapeutic effect on the ALK-positive NSCLC patients, and overcame known ALK resistance mutations, including the most common resistance mutation to the second-generation inhibitors aiming to the ALK G1202R mutant (Yang and Gong, 2019). It also could easily penetrate the blood-brain barriers, which had a benefit for patients with brain metastasis (Johnson et al., 2014). In addition to these secondary resistance mutations, mutations or amplification of bypass signallings (such as AXL, Hh, ERBb2, etc) can also lead to acquired resistance to tyrosine inhibitors (Morgillo et al., 2016).

**FIGURE 1:**
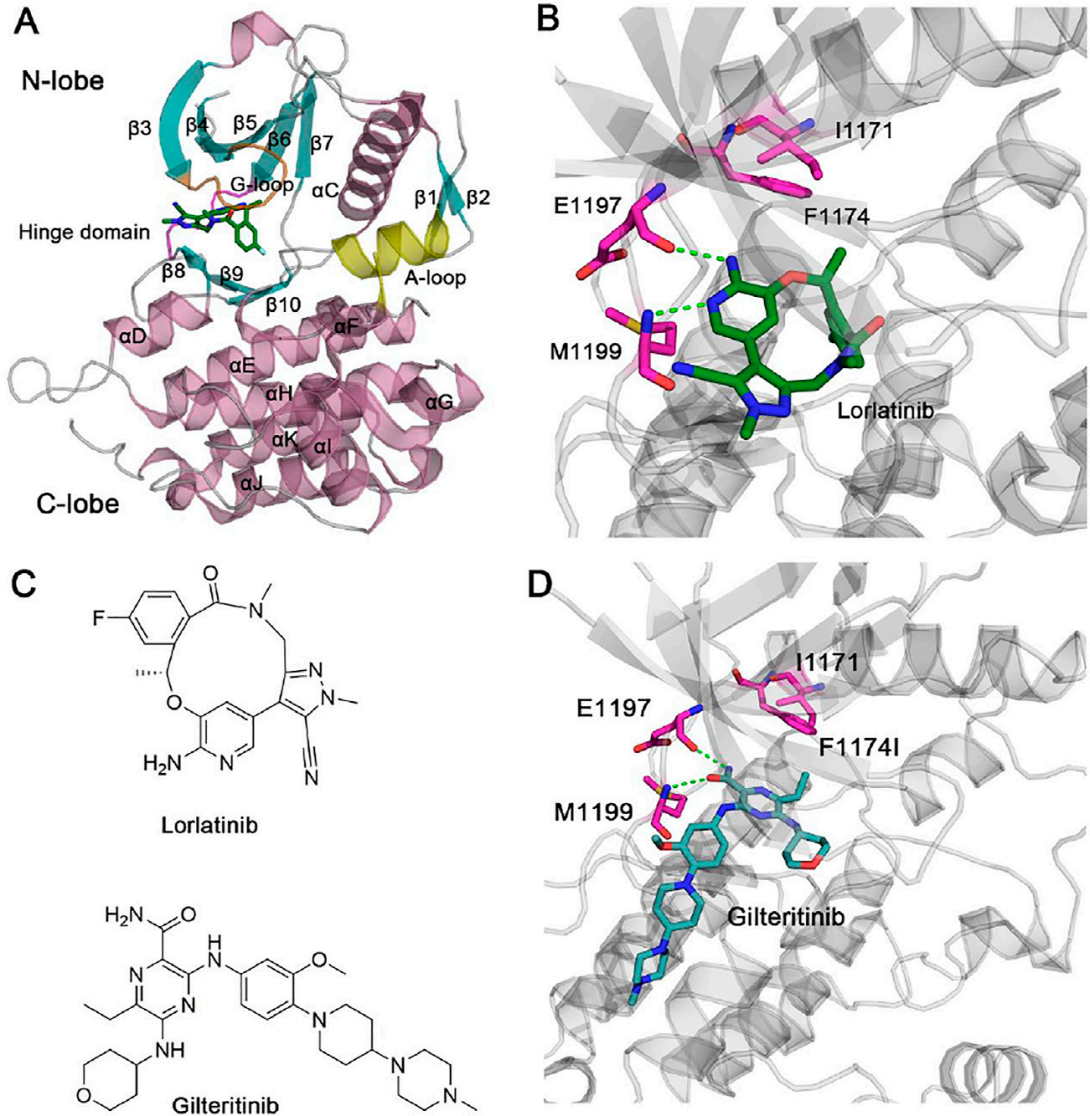
**(A)** X-ray structure of ALK kinase domain in complex with lorlatinib (PDB ID: 4CLI). ALK is shown in cartoon format with the *β*-sheets, *α*-helices, and the loops coloring by cyan, pink, and gray, respectively. The critical glycine-rich loop (G-loop), the hinge domain, and the activation loop (A-loop) are coloring by orange, magenta, and yellow, respectively. **(B)** The detailed hydrogen bonds formed between the hinge residues Glu1197, Met1199 and lorlatinib are depicted by green dotted lines. Residues Ile1171 and Phe1194 are shown by stick models. **(C)** Chemical structures of lorlatinib and gilteritinib. **(D)** The docking pose of ALK in complex with gilteritinib. The detailed hydrogen bonds formed between the hinge residues Glu1197, Met1199 and gilteritinib are depicted by green dotted lines. Residues Ile1171 and Phe1194 are shown by stick models.

The human full-length ALK protein has 1,620 amino acid residues, which consists of four domains, including a signal peptide, an extracellular ligand-binding domain, a transmembrane domain, and an intracellular tyrosine kinase domain. The intracellular tyrosine kinase domain is a targeted position where inhibitors can bind. The kinase domain is composed of a small N-terminal lobe (N-lobe) and a large C-terminal lobe (C-lobe), and the flexible hinge domain connects the two lobes (Figure 1A). ATP molecule or inhibitors are sandwiched by the two lobes under the glycine-rich loop (G-loop) (Figure 1B). The small N-lobe is largely comprised of five-stranded *β*-sheets (*β*3*–β*7) and a catalytically regulatory *α*-helix named the *α*C-helix. The large C-lobe is mainly composed of six conserved *α*-helices (*α*D–*α*I) and two short conserved *β*-strands (*β*9*–β*10). Remarkably, in the unphosphorylated state, the important activation loop (A-loop) forms an additional helix following the *β*10 strand. A conserved D^1270^F^1271^G^1272^ motif is within the A-loop wherein Asp1270 is a critical residue involved in the catalytic activity of ALK to phosphorylate its substrates.

Lorlatinib is a macrocyclic, ATP-competitive inhibitor that binds to the intracellular tyrosine kinase domain (Johnson et al., 2014) (Figure 1C). The X-ray crystal structure of lorlatinib bound to the ALK kinase domain shows that the aminopyridine moiety of macrocycle lorlatinib forms two hydrogen bonds with the backbone moiety of Glu1197 and Met1199 from the flexible hinge domain, respectively (Figure 1B). Such hydrogen-bonding interactions between the hinge residues and inhibitors are conserved in known kinase inhibitors (Attwood et al., 2021; Cohen et al., 2021; Roskoski, 2021). For the structural perspective, Gly1202, Ser1206, and Gly1269 do not interact directly with the lorlatinib and thus, G1202R, S1206Y, and G1269A mutations had minor effects on the therapeutic activity of lorlatinib. However, a double mutation I1171N/F1174I located at the *α*C-helix that is also distant from lorlatinib confers lorlatinib resistance, suggesting that this resistance is through an allosteric regulatory mechanism (Nussinov and Tsai, 2013; Lu et al., 2016; Lu and Zhang, 2019; Lu et al., 2019a; Lu et al.,2019c; Byun et al., 2020; Ni et al., 2020; Ni et al., 2021a). However, the detailed resistance mechanism remains poorly understood.

To overcome the double mutation I1171N/F1174I, a further inhibitor library screening identified a gilteritinib inhibitor that suppressed the viability of both wild-type and the double I1171N/F1174I mutant expressing Ba/F3 cells (Mizuta et al., 2021) (Figure 1C). Currently, the unavailability of a crystal structure of gilteritinib to the ALK kinase domain caused the poor understanding the molecular mechanism of how gilteritinib overcomes lorlatinib resistance in the double mutant ALK I1171N/F1174I.

In this study, the structure model of ALK−gilteritinib complex was first constructed using molecular docking method. Then, multiple replicas of molecular dynamics (MD) simulations of ALK in both wild-type and double I1171N/F1174I mutant were performed in explicit water environment, including ALK^WT^−lorlatinib, ALK^WT^−gilteritinib, ALK^I1171N/F1174I^−lorlatinib, and ALK^I1171N/F1174I^−gilteritinib complexes. Finally, the effect of double mutation on the conformational dynamics, binding affinity, and interaction mode of lorlatinib- and gilteritinib-bound ALK was examined. The results shed light on the mechanism of gilteritinib overcoming lorlatinib resistance in the double mutant ALK I1171N/F1174I and will help in the future design of next-generation ALK inhibitors.

## Materials and Methods

### Molecular Docking

The starting coordinates of ALK^WT^−lorlatinib complex (PDB ID: 4CLI) were downloaded from the PDB (http://www.rcsb.org) (Johnson et al., 2014). The 4CLI structure was modified for docking study. Lorlatinib was removed from the 4CLI structure and the remaining *apo* protein was used for docking of gilteritinib into the active site using the AutoDock 4.2 program (Morris et al., 2009). Polar hydrogen atoms were added to the ALK using the Hydrogen module in AutoDock Tools (ADT), Kollman united atom partial charges were then added to the ALK, and the AutoDock atom type were defined for the ALK using ADT. For the gilteritinib, all hydrogen atoms were added and the root, rotatable bonds, as well as torsion of the inhibitor were set using the default values. Docking was carried out using the protein kept rigid, whereas the inhibitor was allowed to move freely and with a docking box covering the cavity of the active site of ALK (Jung et al., 2019; Kang et al., 2020; Choi et al., 2021). The Lamarckian genetic algorithm was used for the conformational search of the inhibitor within the docking box size. During docking process, 100 independent runs were performed and the resulting poses were clustered using a root-mean-square deviation (RMSD) cutoff of 1 Å (Ma et al., 2020; Wu et al., 2020). The docking pose with the lowest energy in the largest cluster was visually analyzed and then selected for the following MD simulations.

### MD Simulations

The two mutant systems, ALK^I1171N/F1174I^−lorlatinib and ALK^I1171N/F1174I^−gilteritinib, were constructed based on the corresponding wild-type structural complex by replacing target residues with the desired amino acid residues using the Discovery Studio program. The two missing disorders loops (Ser1136–Ser1143 and Ala1280–Lys1285) were modelled using the Modeller v9.16 (Fiser and Sali, 2003). The simulations were performed using the AMBER 16 package (Case et al., 2005). The Amber ff14SB force field was assigned for the protein and ions (Maier et al., 2015) and the general amber force field (GAFF) was applied for the inhibitors (Wang et al., 2004). The four protein-ligand complexes were solvated in a commonly used TIP3P water box (Jorgensen et al., 1983) and counterions were then added to neutralize the systems. A total of 0.15 mol/L NaCl was added to the solvent to represent the physiological condition.

The four systems were performed with two rounds of energy minimization as reported previously by a combination of steepest descent and conjugate gradient minimizations (Lu et al., 2019b; Mahalapbutr et al., 2020; Shi et al., 2020; Vatansever et al., 2020; Wang et al., 2021a; Lu et al., 2021b). Afterwards, 500 ps heating, and 1,000 ps equilibration at 300 K under the *NVT* ensemble were performed with all heavy atoms of protein-ligand complexes fixed by a 10 kcal/(mol Å^2^) force constant. Finally, 15 independent replicas of 1,000 ns simulations for each system were performed with random velocities under the *NPT* ensemble, generating a total of 60 μs simulated trajectories. The particle mesh ewald (PME) method (Darden et al., 1993) was used to calculate long-range electrostatic interactions and the SHAKE algorithm (Ryckaert et al., 1977) was used to constrain all covalent bonds involving hydrogen atoms. The temperature and pressure were coupled with a time constant of 1.0 ps using the Langevin’s algorithm (Wu and Brooks, 2003). An integration time step of 2 fs was used.

### Principal Component Analysis

Principal component analysis (PCA) is a useful method to show slow motion dynamics of proteins, named essential dynamics (Rehman et al., 2019; Neves Cruz et al., 2020; Rehman et al.,2021). According to PCA, the covariance matrix of Cα atoms was diagonalized to generate a set of eigenvalues and the corresponding eigenvectors. Each eigenvector also called principal component (PC) was related to an eigenvalue corresponding to the mean square fluctuation projected along the that eigenvector. The first several PCs constitute largely the overall fluctuations of proteins. In the present study, each snapshot sampled during MD simulations was projected into the collective coordinate space defined by the first two eigenvectors (PC1 and PC2), representing the essential conformational subspace sampled by different ALK states.

### Binding Free Energy Calculations

The molecular mechanisms generalized Born surface area (MM-GBSA) energy calculations were performed using the following equation (Hou et al., 2011; Xie et al., 2019; Zeb et al., 2019; Khan et al., 2020; Li et al., 2020a):

$$\Delta G_{binding}=\Delta G_{complex}-\left(\Delta G_{protein}+\Delta G_{ligand}\right)$$ (1)

$$\Delta G_{binding}=\Delta E_{gas}+\Delta G_{solvation}-T\Delta S$$ (2)

$$\Delta E_{gas}=\Delta E_{vdW}+\Delta E_{ele}$$ (3)

$$\Delta G_{solvation}=\Delta G_{GB}+\Delta E_{nonpolar}$$ (4)

$$\Delta G_{nonpolar}=\gamma \times SASA+b$$ (5)

Wherein Δ*E*_gas_, Δ*E*_vdW_, Δ*E*_ele_, Δ*G*_*s*olvation_, Δ*G*_GB_, and Δ*G*_nonpolar_ represented gas energy, van der Waals energy, electrostatic energy, solvation free energy, the polar energy, and the nonpolar energy, respectively. The Δ*G*_GB_ was calculated using the GB model (Onufriev’s GB, IGB = 2) (Kollman et al., 2000). The Δ*G*_nonpolar_ was calculated using the function of the solvent accessible surface area (SASA) with the γ value of 0.0072 kcal/(mol Å^2^) and the b value of 0 kcal/mol. The *T*Δ*S* was not computed in this work because of the extremely long durations of normal mode analysis for large systems (Wang et al., 2019; Yang et al., 2020).

Residue free energy calculation was carried out to reveal the critical residues responsible for inhibitor binding by dividing the total free energy into the energy contributions from individual protein and inhibitor interaction pairs using the above MM-GBSA method.

### Generalized Correlation (*GC*_*ij*_) Analysis

The generalized correlation (*GC*_*ij*_) analysis illustrated independent correlations on the relative orientation of the atomic fluctuations, which can unravel non-linear correlations (Palermo et al., 2016; Saha et al., 2020; Li et al., 2021a). In this analysis, two variables were regarded correlated when the product of their marginal distribution $p\left(x_{i}\right)\cdot p\left(x_{j}\right)$ was larger than their joint distribution $p\left(x_{i},x_{j}\right)$ . To measure the degree of correlation between selected variables, mutual information (*MI*) between $x_{i}$ and $x_{j}$ was calculated as:

$$\begin{array}{c} MI\left[x_{i},x_{j}\right]=\iint p\left(x_{i},x_{j}\right)ln\frac{p\left(x_{i},x_{j}\right)}{p\left(x_{i}\right)\cdot p\left(x_{j}\right)}dx_{i}dx_{j} \end{array}$$ [6]

where the equation [6] defined *MI* as closely related to the well-known Shannon entropy H[x] , which was calculated as:

$$\begin{array}{c} H\left[x\right]=\int p\left(x\right)lnp\left(x\right)dx \end{array}$$ [7]

The correlation between pairs of atoms $x_{i}$ and $x_{j}$ was described by *MI* and calculated using the marginal Shannon entropy $H\left[x_{i}\right]$ , $H\left[x_{j}\right]$ , and the joint entropy term $H\left[x_{i},x_{j}\right]$ :

$$\begin{array}{c} MI\left[x_{i},x_{j}\right]=H\left[x_{i}\right]+H\left[x_{j}\right]-H\left[x_{i},x_{j}\right] \end{array}$$ [8]

The g_correlation tool within the GROMACS 4.6 package (Abraham et al., 2015) was used to compute the entropy terms $H\left[x_{i}\right]$ , $H\left[x_{j}\right]$ , and $H\left[x_{i},x_{j}\right]$ with the *k*-nearest neighbour distance algorithm using atomic fluctuation information. The $MI\left[x_{i},x_{j}\right]$ values were further normalised to obtain the normalised generalised correlation coefficients ( $GC_{ij}$ ):

$$\begin{array}{c} GC_{ij}={\left\{1-e^{-\frac{2MI\left[x_{i},x_{j}\right]}{d}}\right\}}^{\frac{1}{2}} \end{array}$$ [9]

where d represented the dimensionality of $x_{i}$ and $x_{j}$ .

### Cross-Correlation (*CC*_*ij*_) Analysis

The cross-correlation matrix (*CC*_*i*j_) based on Pearson coefficients between the fluctuations of the Cα atoms relative to their average positions was used to uncover the coupling of the motions between the protein residues (He et al., 2021; Lu et al., 2021a; Wang et al., 2021b). *CC*_*ij*_ was calculated using following equation,

$$C\left(i,j\right)=\frac{c\left(i,j\right)}{c{\left(i,i\right)}^{1/2}c{\left(j,j\right)}^{1/2}}$$ [10]

Positive *CC*_*ij*_ values mean the two atoms *i* and *j* moving in the same direction, whereas negative *CC*_*ij*_ values describe anti-correlated motions between the two atoms *i* and *j.*

## Results and Discssion

### Modeling of ALK−Gilteritinib Complex

Gilteritinib is an ATP-competitive tyrosine multi-kinase inhibitor that has been approved for treating relapsed or refractory Fms-like tyrosine kinase 3 (FLT3)-positive acute myeloid leukemia (AML) (Pulte et al., 2021). Multiple experiments containing the ALK kinase activity, phosphoproteomic analysis of ALK-positive lung cancer cells, and kinase substrate-enrichment analysis unequivocally ascertained that gilteritinib directly inhibited the growth of *ALK*-rearranged human NSCLC cells and further overcame lorlatinib resistance to the double mutant ALK I1171N/F1174I (Mizuta et al., 2021). These data indicated that in addition to treat relapsed or refractory FLT3-positive AML, gilteritinib could also function as drug repositioning, that is, treating for ALK-positive NSCLC including lorlatinib-resistant ALK I1171N/F1174I double mutant.

Up to now, an X-ray crystal structure of gilteritinib complexed with the ALK kinase domain has not been resolved, rendering the detailed binding mode between the ALK active site and gilteritinib still unknown. However, the X-ray crystal structure of gilteritinib bound to the FLT3 kinase domain is now available (PDB ID: 4JQR) (Kawase et al., 2019). The gilteritinib was extracted from the 4JQR structure, and we then employed molecular docking method to dock gilteritinib into the ALK active site using the crystal structure of ALK–lorlatinib complex (PDB ID: 4CLI) (Johnson et al., 2014). Molecular docking method has been widely used to model previously unknown protein kinase/enzyme–ligand interactions such as epidermal growth factor receptor (EFGR)–osimertinib (Qiu et al., 2021), angiotension-converting 2 (ACE2)–puerarin/quercetin (Pan et al., 2020), proliferator activated receptor γ (PPARγ)–bavachinin (Feng et al., 2021), and sirtuin 6 (SIRT6)–JYQ-42 interactions (Zhang et al., 2021).

Figure 1D shows the docking pose of ALK–gilteritinib complex that has the lowest energy extracted from the largest cluster. In the ATP-binding site, the amide imidazole moiety is involved in two hydrogen bonds with the backbone moiety of Glu1197 and Met1199 from the flexible hinge domain, respectively. This conserved hydrogen-bonding interactions between the ALK hinge residues and gilteritinib are also observed in the crystal structural complex of ALK–lorlatinib (Johnson et al., 2014). The tetrahydropyran moiety interacts with the G-loop residues and the terminal methyl piperazine moiety protrudes into the solvent. The closest heavy atomic distance between gilteritinib and the nearby Ile1171 is approximately 6.0 Å. In contrast, the closest heavy atomic distance between lorlatinib and Ile1171 is 7.2 Å, which is larger than that in the ALK–gilteritinib complex. However, the underlying mechanism of how gilteritinib overcomes lorlatinib resistance to the double mutant I1171N/F1174I is incapable of elucidating directly based on the structural comparison of ALK–lorlatinib and ALK–gilteritinib complexes. To address this issue, MD simulations that consider conformational dynamics of proteins were performed to illuminate the effect of double mutations I1171N/F1174I on the conformational plasticity of ALK–lorlatinib and ALK–gilteritinib complexes (Sora et al., 2020; Wang, et al., 2020; Zhou et al., 2021; Li et al., 2021b; Ni et al., 2021b).

### Double Mutations Had a Minor Effect on the ALK Kinase Domain Conformational Dynamics

Previously, a large amount of *in silico* studies containing MD simulations and binding free energy calculations were performed to provide the mechanism of drug resistance conferred by the clinically acquired mutants of ALK. These studies included the revelation of the drug-resistance mechanism of crizotinib to the C1156Y (Sun and Ji, 2012; Chen et al., 2020a), F1174L (Kumar and Ramanathan, 2014), F1174V (Dehghanian et al., 2017), L1196M (Kay and Dehghanian, 2017; Nagasundaram et al., 2017), L1198F (Li et al., 2017; Chuang et al., 2019; Chen et al., 2020a), G1202R (Chuang et al., 2019), S1206C (Li et al., 2018), G1269A (Nagasundaram et al., 2017), and C1156Y/L1198F (Chen et al., 2020a), ceritinib to the G1123S (He et al., 2019), I1171T (Ni and Zhang, 2015), F1174C (Ni et al., 2016), and G1202R (Chen et al., 2018), alectinib to the G1202K (Yang et al., 2021), I1171N, V1180L, and L1198F (He et al., 2018), and G1202R (Wang et al., 2018), and lorlatinib to the I1171N and G1202R (Okada et al., 2019). The main findings from above results were obvious because the vast majority of mutations are located at the ATP-binding site and these mutations would disturb the ALK−inhibitor interactions. However, in our present study, the double mutations I1171N/F1174I do not make direct contacts with both lorlatinib and gilteritinib, and thus the drug resistant mechanism cannot be directly deduced from the structural complexes. As such, we performed μs-length MD simulations to propagate the perturbations from the mutated site to the inhibitor-binding site.

We explored ALK^WT^−lorlatinib, ALK^WT^−gilteritinib, ALK^I1171N/F1174I^−lorlatinib, and ALK^I1171N/F1174I^−gilteritinib complexes to reveal similarities and differences in the conformational dynamics across various states (i.e., lorlatinib-bound vs gilteritinib-bound, and wild-type vs double mutant). For each system of ALK, MD simulations were performed in explicit water environment, generating multiple μs-length trajectories (i.e., 15 replicas of 1 μs for each system) and yielding an accumulated sampling of 60 μs. These simulating multiple and independent μs-length trajectories were required to achieve solid statistics for the analysis of different dynamics of ALK, because multiple ns-to-μs MD trajectories are essential to reveal the interdependent dynamics of protein domains and their interactions with the inhibitors (Anggayasti et al., 2020; Jang et al., 2020; Liang, et la., 2020; Navarro et al., 2020; Shevchenko et al., 2020; Shibata et al., 2020; Li et al., 2020b).

To show the global flexibility of the wild-type and double mutant ALK when bound to lorlatinib and gilteritinib, root-mean-square fluctuation (RMSF) calculation was first carried out. RMSF analysis is a conventional index to assess protein plasticity. As shown in Supplementary Figure S1, the RMSF plot revealed high fluctuations of the G-loop, the *β*-turn that connecting *β*4 to *β*5, the disordered loop that connecting *α*D to *α*E, the A-loop, and the two N- and C-terminal loops, which were conserved along the simulated runs independent of wild-type and mutant systems. The SEM error bars were shown with respect to average structure of the respective state. Significantly, the large changes were observed in the conformation of the A-loop in all runs, suggesting the conformational flexibility of the A-loop. This is notable because that the conformation of the A-loop plays a critical role in modulating the kinase catalytic activity (Pearce et al., 2010). On the other hand, the RMSF of the *β*-sheets and the *α*-helices exhibited low fluctuations. This was consistent with high structural stability of these ordered domains, also observed in MD simulations of other tyrosine protein kinases. Further assessment of the RMSD of the protein C*α* atoms showed that the conformational dynamics of ALK kinase domain behaved a similar stability in both lorlatinib- and gilteritinib-bound states regardless of wild-type or mutant systems (i.e., the RMSD reached ∼2 Å, Supplementary Figure S2). Taken together, these data revealed that upon lorlatinib or gilteritinib binding to the ATP-binding site of the ALK kinase domain, the overall stability of the protein was preserved in both the wild-type and mutant systems, implying that the double mutations had a minor effect on the overall conformational dynamics of ALK with different inhibitor-bound states.

### Double Mutations Quenched the Dynamics of ALK

To uncover the large-scale collective motions of the ALK−inhibitor complexes and the conformational interconversion of the protein through different states, we performed principal component analysis (PCA) of the four simulated systems. Based on the PCA analysis, the directionality and amplitude of protein motions, in which the first several principal components (i.e., principal components 1 and 2, PC1 and PC2) are associated with the large conformational changes of the complexes. PCA has been successfully applied to decipher experimentally observed conformational variations of proteins (Masterson et al., 2011). In the present study, we combined the collected trajectories and subsequently subjected to RMS-fit to the initial crystal structure of ALK−lorlatinib complex as the same reference configuration. This operation ensured consistency of the motions of the principal components.

**FIGURE 2:**
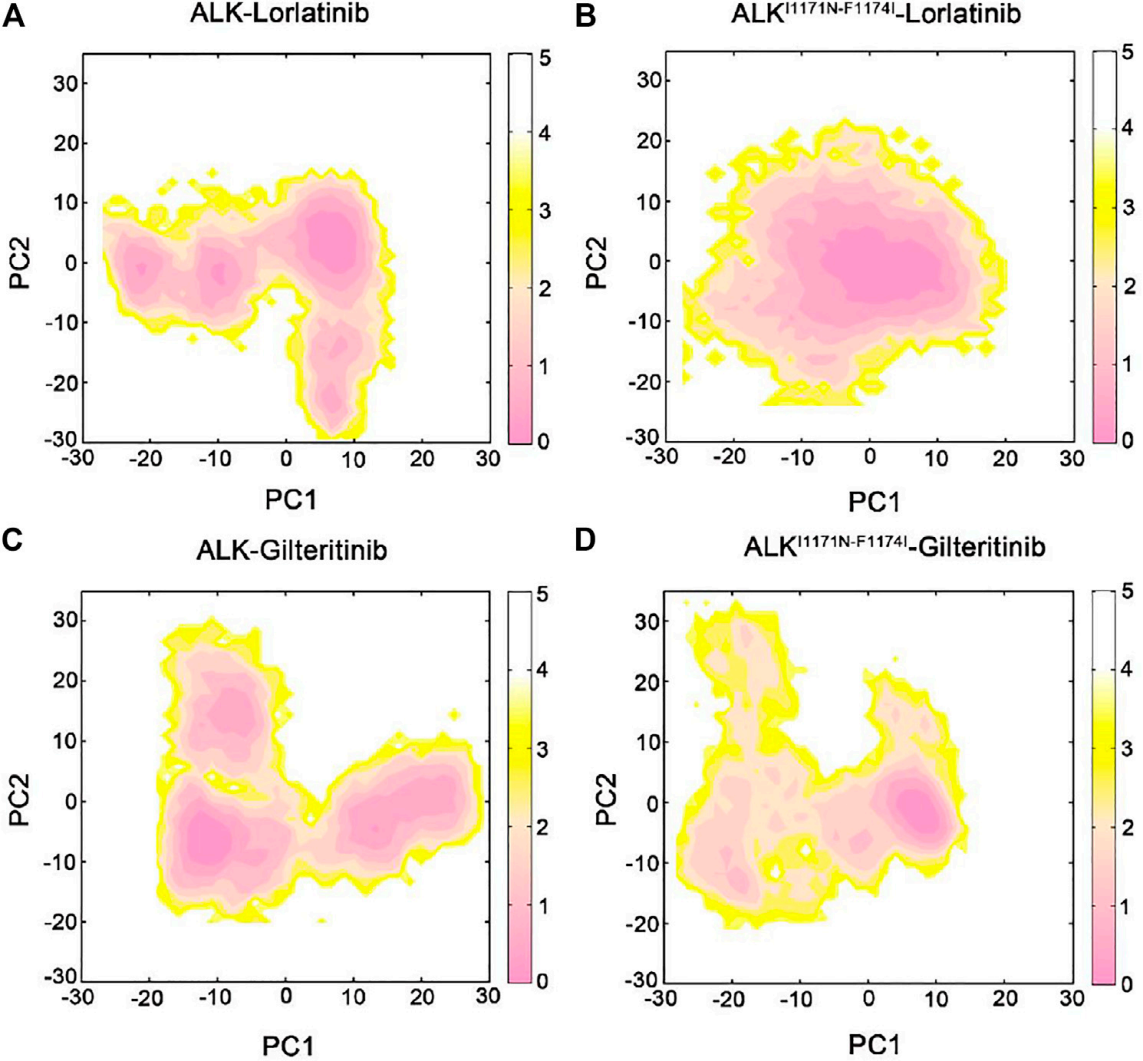
The free energy landscape of the first and second principal components (PC1 vs PC2) from MD simulations of the ALK–lorlatinib **(A)**, ALK^I1171N−F1174I^–lorlatinib **(B)**, ALK–gilteritinib **(C)**, and ALK^I1171N−F1174I^–gilteritinib **(D)**. The unit of free-energy values is kcal/mol.

We executed the PCA analysis for the ALK kinase domain in complex with lorlatinib and gilteritinib in both wild-type and double mutant systems and observed that the first two components (PC1 and PC2) represented ∼65% of variance in coordinates along the MD simulations. Figure 2 showed the free energy landscapes of the PC1 and PC2 that could characterize the conformational space adopted by the different ALK states. In both the wild-type ALK−lorlatinib (Figure 2A) and ALK−gilteritinib (Figure 2C) complexes, the PC1 vs PC2 plots sampled a broad distribution of conformations and identified several conformational states. For example, the PC1 and PC2 values in the wild-type ALK−lorlatinib are calculated from ∼−30 to ∼10, and from ∼−30 to ∼10, respectively (Figure 2A). The PC1 and PC2 values in the wild-type ALK−gilteritinib are calculated from ∼−20 to ∼30, and from ∼−20 to ∼25, respectively (Figure 2C). Futhermore, we used the cluster analysis to extract the most representative conformation from each of three clusters in both wild-type lorlatinib- and gilteritinib-bound states. The conformational superimposition showed that the two critical flexible loops, the G-loop and the A-loop, exhibited large conformational changes in both systems (Supplementary Figure S3). However, in the double mutant I1171N/F1174I ALK−lorlatinib (Figure 2B) and ALK−gilteritinib (Figure 2D) complexes, the PC1 vs PC2 plots sampled a limited distribution of conformations with the PC1 from ∼−10 to ∼10 and the PC2 from ∼−10 to ∼5, respectively, and identified one major conformational state. This result indicated that the double mutations quenched the dynamics of ALK and a restriction of the conformational space of ALK was sampled upon double mutations. However, by comparing the free-energy surface of the double mutant, we found that the free-energy basin was more restricted in the ALK−gilteritinib complex (Figure 2D) than in the ALK−lorlatinib complex (Figure 2B). This further suggested that the formation of ALK−inhibitor interactions was more stable in the gilteritinib-bound than the lorlatinib-bound states in response to double mutations.

### Coupled Motions of Protein Domains

In order to explore the interdependent conformational dynamics of the ALK kinase domain among spatially distinct domains in different states, dynamic correlation analysis was carried out. We used two different methods to calculate the dynamic correlated motions of protein domains, including the traditional Pearson cross-correlation (CC*ij*) coefficients and the generalized correlation (GC*ij*) coefficients. The CC*ij* index computes the collinear correlation between the 2 C*α* atoms (*i* and *j*), showing whether they move in the correlated (CC*ij* > 0) motions or in the anti-correlated (CC*ij* < 0) motions. The analysis of CC*ij* is solely based on correlations that are collinear with each other, discarding correlated motions that are out of phase. By contrast, the GC*ij* analysis computes the degree of correlation between the 2 C*α* atoms using their mutual information. This coefficient yields a normalized assessment of how much information on 1 C*α* atom is offered by that of another C*α* atom. Yet, the GC*ij* coefficient is incapable of distinguishing correlated or anti-correlated motions between the 2 C*α* atoms. As a result, when combined, the CC*ij* and GC*ij* coefficients are useful to elucidate the interdependent dynamics of proteins.

**FIGURE 3:**
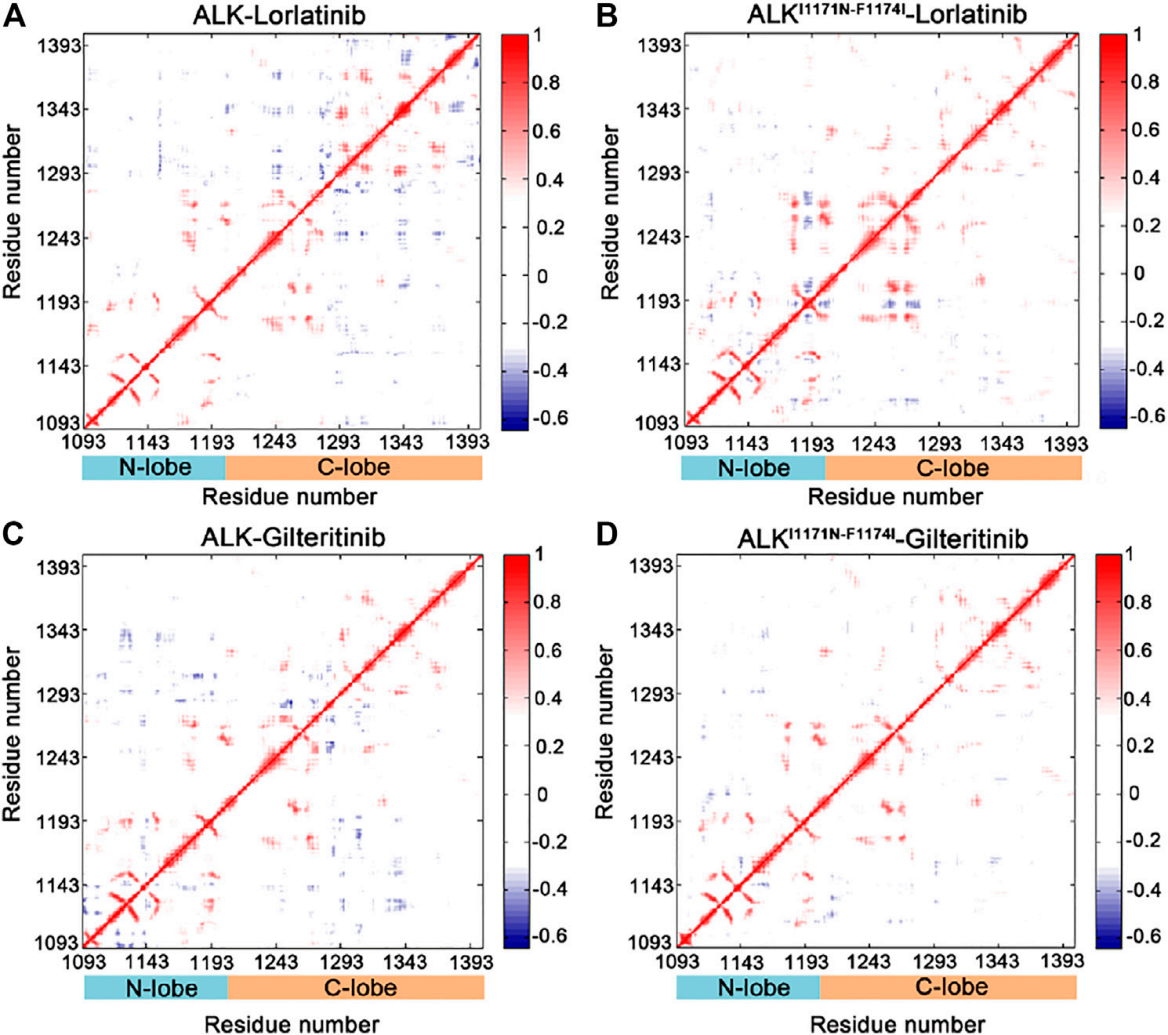
Cross-correlation (CC_*ij*_) matrix computed for the ALK–lorlatinib **(A)**, ALK^I1171N−F1174I^–lorlatinib **(B)**, ALK–gilteritinib **(C)**, and ALK^I1171N−F1174I^–gilteritinib **(D)**. The correlated motions are colored by red (CC_*ij*_ > 0), while the anti-correlated motions are colored by blue (CC_*ij*_ < 0). Color scales are shown at the right. The CC_*ij*_ values with an absolute correlation coefficient of less than 0.3 are colored by white for clarity.

The CC*ij* matrix of ALK kinase domain exhibited a conserved pattern of correlated and anti-correlated motions in both wild-type (Figure 3A) and double mutant (Figure 3B) lorlatinib-bound states as well as in wild-type (Figure 3C) and double mutant (Figure 3D) gilteritinib-bound states. However, in both the wild-type lorlatinib- and gilteritinib-bound states, the N-lobe (residues 1,093–1,203) showed enhanced anti-correlated motions with the C-lobe (resdues 1,204–1,401) compared to both the double mutant states (Supplementary Figure S4). This pattern of the anti-correlated motions between the N- and C-lobes has been previously reported in MD simulations of other protein kinases such as protein kinase A (Masterson et al., 2011), glycogen synthase kinase 3β (Lu et al., 2011), and EGFR (Qiu et al., 2021). Collectively, the CC*ij* analysis indicated that the double mutations I1171N/F1174I decreased the anti-correlated motions between the N- and C-lobes of ALK kinase domain.

**FIGURE 4:**
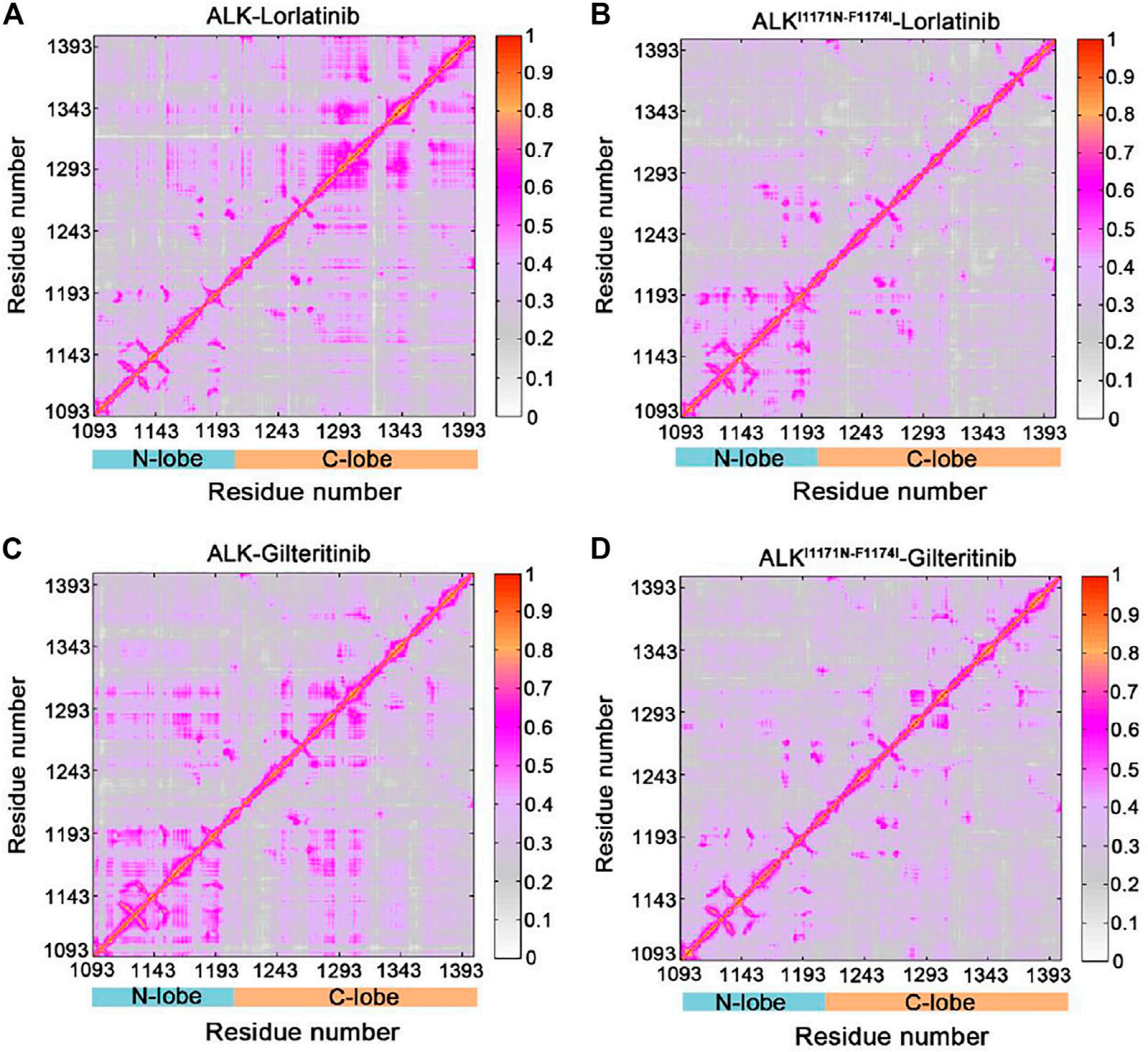
Generalized correlation (GC_*ij*_) matrix computed for the ALK–lorlatinib **(A)**, ALK^I1171N−F1174I^–lorlatinib **(B)**, ALK–gilteritinib **(C)**, and ALK^I1171N−F1174I^–gilteritinib **(D)**. Color scales are shown at the right.

**FIGURE 5:**
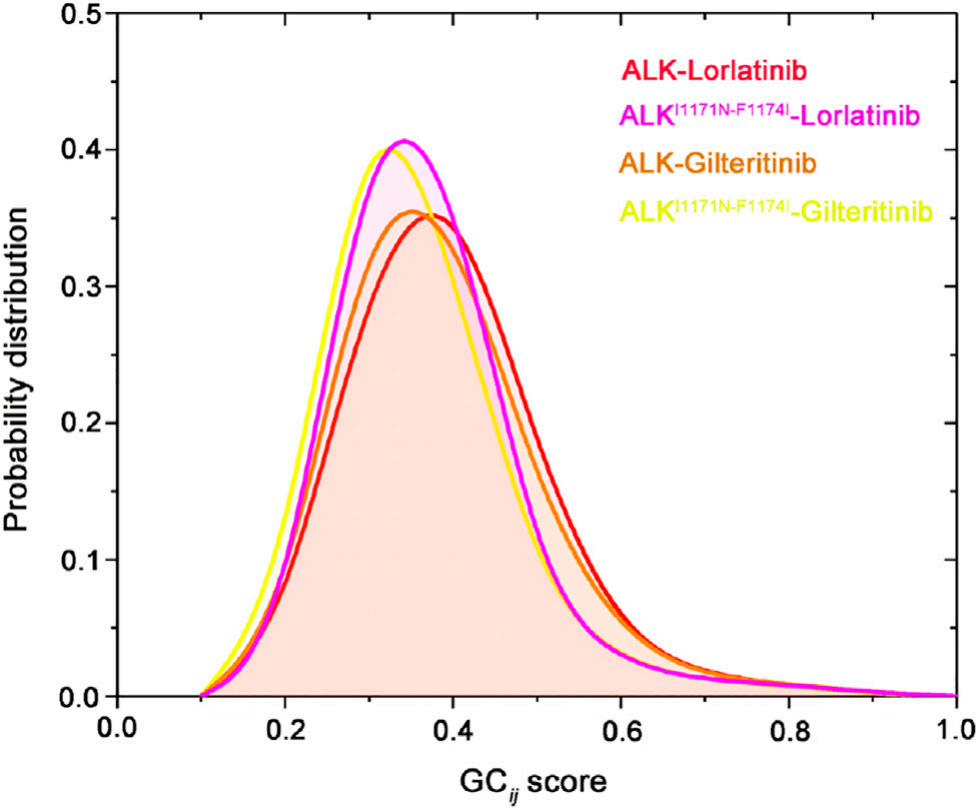
Probability distribution of the normalized GC*ij* scores for the four ALK states.

The GC*ij* matrix can uncover the global dependencies of the protein motions (Figure 4). Similarly, the wild-type lorlatinib-bound (Figure 4A) and gilteritinib-bound (Figure 4C) ALK showed a higher degree of correlations between the N-lobe and the C-lobe compared to the double mutant lorlatinib-bound (Figure 4B) and gilteritinib-bound (Figure 4D) states. This result implied a shift in the globally correlated motions of protein domains upon double mutations. To quantitatively assess the interdependent coupling between protein domains in different ALK states, we calculated the average GC*ij* scores, which accumulated and normalized the GC*ij* for the whole residues. Figure 5 showed the probability distributions of the normalized GC*ij* scores for the four ALK states. Notably, both the wild-type ALK−lorlatinib (∼0.4) and ALK−gilteritinib (∼0.36) complexes had a higher GC*ij* score than their respective double mutant systems (∼0.3 for ALK^I1171N−F1174I^−gilteritinib and ∼0.32 for ALK^I1171N−F1174I^−lorlatinib). Furthermore, both the wild-type systems had a more distribution of large GC*ij* scores in the range of 0.5–0.7 compared to both the double mutant systems. This result indicated that the double mutations reduced the correlation motions of interdependent domains, which was consistent with the PCA analysis.

### Binding Free Energy Calculations

To further evaluate the impact of double mutations on the binding abilities of ALK−lorlatinib and ALK−gilteritinib complexes, the binding free energies for the four systems were calculated using the MM/GBSA method using the 500 snapshots that were equally extracted from the last 300 ns MD trajectories. As show in Table 1, the predicted binding free energies (Δ*G*_binding_) for ALK−lorlatinib, ALK^I1171N−F1174I^−lorlatinib, ALK−gilteritinib, and ALK^I1171N−F1174I^−gilteritinib are −39.47 ± 2.91, −31.80 ± 3.99, −39.21 ± 5.65, and −37.14 ± 5.56 kcal/mol, respectively. That is to say, in the wild-type system, lorlatinib and gilteritinib bound to the ALK kinase domain with a similar ability. This prediction was in consistent with the experimental CellTiter-Glo assays that the IC_50_ value of lorlatinib (1.2 ± 0.38 nM) and gilteritinib (0.78 ± 0.27 nM) to ALK was nearly the same, suggesting the equal inhibitory capacity of ALK by lorlatinib and gilteritinib (Mizuta et al., 2021). In the double mutant of gilteritinib-bound state, the predicted binding free energy was slightly higher by 2.07 kcal/mol than that of the counterpart wild-type system. Indeed, the experimental IC_50_ of gilteritinib to the double mutant I1171N/F1174I was 24 ± 4.4 nM, which increased by ∼30-fold compared to the wild-type ALK (Mizuta et al., 2021). Significantly, when lorlatinib bound to the double mutant, the predicted binding free energy caused an increase of 7.67 kcal/mol in relation to the corresponding wild-type system. Consistently, the experimental IC_50_ of lortatinib to the double mutant I1171N/F1174I was 338 ± 41 nM, which increased by ∼282-fold compared to the wild-type ALK (Mizuta et al., 2021). Therefore, according to the energetical prediction results, gilteritinib could still bind to the double mutant ALK I1171N/F1174I, albeit with a relatively decreased binding affinity respective to the wild-type state, whereas the double mutations had a highly detrimental effect on the ability of ALK to bind lorlatinib. This would cause lorlatinib resistance to the double mutant. It was worth noting that, for the lorlatinib, the difference in the binding affinities towards the wild-type and the double mutant was largely derived from the difference in the electrostatic interactions (Δ*E*_ele_) and the van der Waals interactions (Δ*E*_vdW_) as shown in Table 1.

**TABLE 1:** Binding free energy (kcal/mol) between the larlatinib/gilteritinib and ALK in both wild-type and double mutant states.

Energy items	Larlatinib-bound ALK		Gilteritinib-bound ALK	
	Wild-type	I1171N-F1174I	Wild-type	I1171N-F1174I
Δ*E*_ele_	−13.98 ± 3.12	−10.98 ± 3.87	−15.07 ± 4.28	−16.27 ± 4.16
Δ*E*_vdW_	−47.77 ± 3.00	−41.79 ± 3.36	−50.36 ± 3.86	−50.40 ± 4.24
ΔG_nonpolar_	−5.71 ± 0.26	−5.27 ± 0.41	−6.38 ± 0.47	−6.21 ± 0.49
ΔG_polar_	27.98 ± 2.75	26.24 ± 4.40	31.03 ± 3.66	34.77 ± 4.19
ΔG_sol_	22.27 ± 2.64	20.97 ± 4.14	24.65 ± 3.50	28.55 ± 4.02
Δ*G*_binding_	−39.47 ± 2.91	−31.80 ± 3.99	−39.21 ± 5.65	−37.14 ± 5.56

### Critical Residues for Binding Specificity Calculated by Free Energy Decomposition and Key Hydrogen Bonds Analysis

**FIGURE 6:**
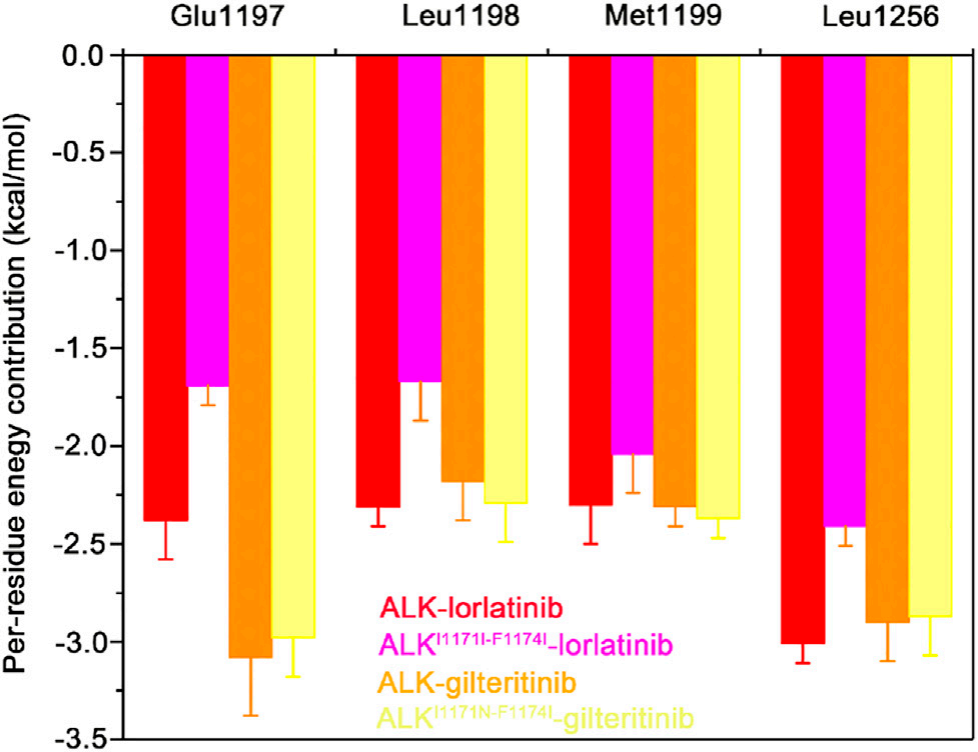
The mainly distinct residue contributions to the binding affinities of lorlatinib/gilteritinib to both the wild-type and double mutant predicted by the MM-GBSA binding free energy decomposition. The error bars represent standard deviations of per-residue energetic contribution.

**FIGURE 7:**
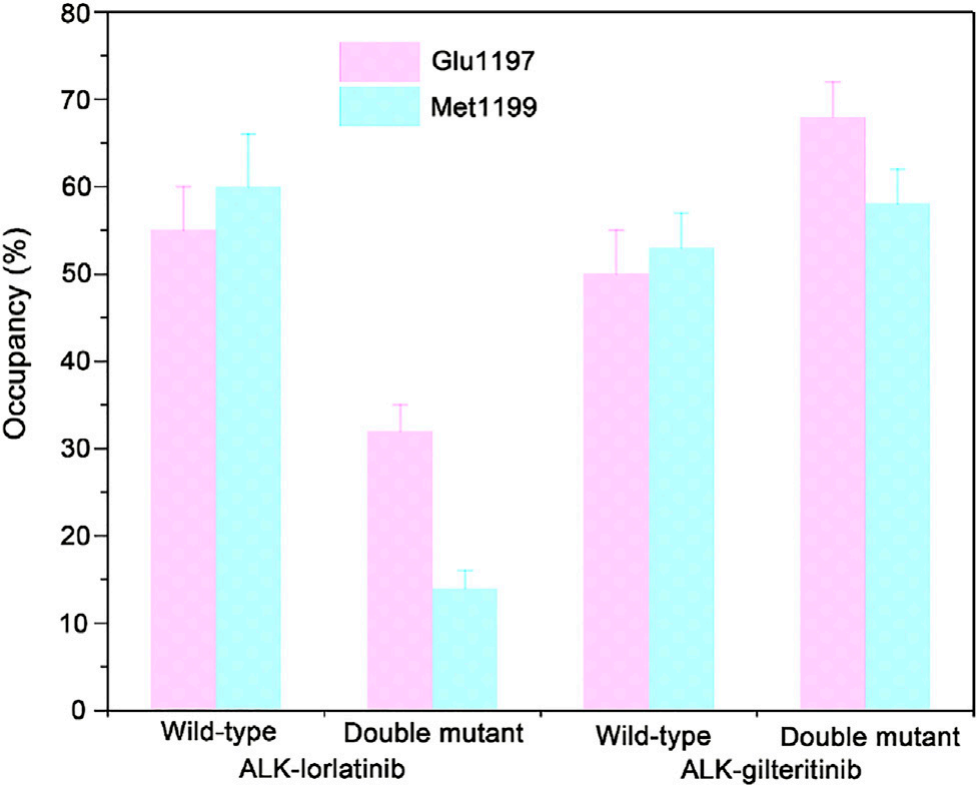
The critical hydrogen bonding interactions between the hinge Glu1197, Met1199 and lorlatinib/gilteritinib in both the wild-type and the double mutant systems. The error bars represent standard deviations of the occupancy of hydrogen bonds.

To further show the critical residues that control the different binding abilities of lorlatinib and gilteritinib to the wild-type and the double mutant systems, the residue-specific binding free energies between lorlatinib/gilteritinib and wild-type/double mutant protein were predicted by the MM-GBSA free energy decomposition analysis. The total binding free energy was decomposed and the top differential residues with the energetical contributions to inhibitor binding were selected. As shown in Figure 6, it can be found that Glu1197, Leu1198, Met1199, and Leu1256 are the most important residues that provides distinct binding contributions between the wild-type and double mutant ALK−lorlatinib complexes, whereas these residues exhibit similar contributions between wild-type and double mutant ALK−gilteritinib complexes. Obviously, the Glu1197, Leu1198, Met1199 are located at the hinge domain with the formation of two hydrogen bonds between Glu1197, Met1199 and lorlatinib based on the crystal structural complex. The Leu1256 located at the base of the ATP-binding site forms van der Waals interactions with the lorlatinib. Based on the decomposition free energy analysis, we hypothesized that the double mutations I1171N/F1174I would disturb the hydrogen bonding interactions between Glu1197, Met1199 and lorlatinib. To test this hypothesis, we then analyzed the distributions of the hydrogen bonds formed between the ALK kinase domain and lorlatinib/gilteritinib in both the wild-type and double mutant systems along the MD simulations. As shown in Figure 7, the formation of two hydrogen bonds between Glu1197, Met1199 and the inhibitor was conserved in the three simulated systems, including the wild-type ALK−lorlatinib, wild-type ALK−gilteritinib, and ALKI^1171N−F1174I^−gilteritinib complexes. In sharp contrast, in the ALK^1171N−F1174I^−lorlatinib complex, the occupancy of the two hydrogen bonds was reduced upon the double mutations I1171N/F1174I. Thus, the disruption of the key hydrogen bonds between the hinge residues and lorlatinib due to the double mutations of ALK might uncouple the lorlatinib to the double mutant.

### Comparison of Binding Modes

**FIGURE 8:**
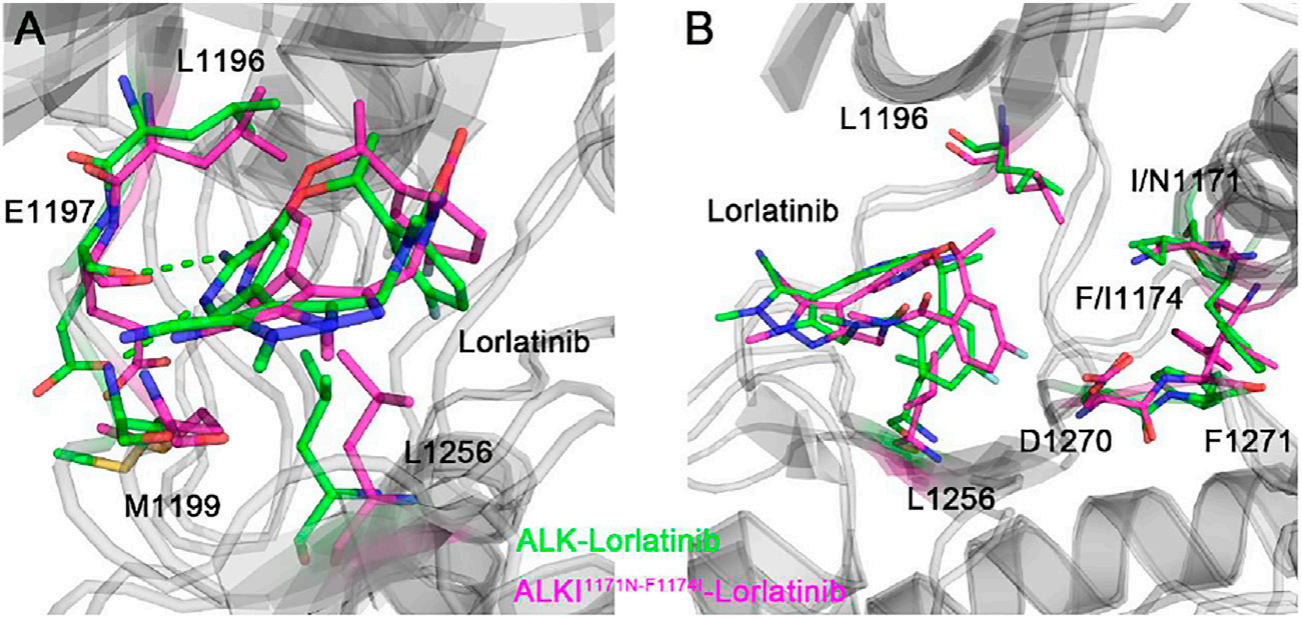
The backbone superimposition of the representative conformation of double mutant I1171N/F1174I lorlatinib-bound ALK to the wild-type structural complex in the ATP-binding site **(A)** and the mutated site **(B)**.

**FIGURE 9:**
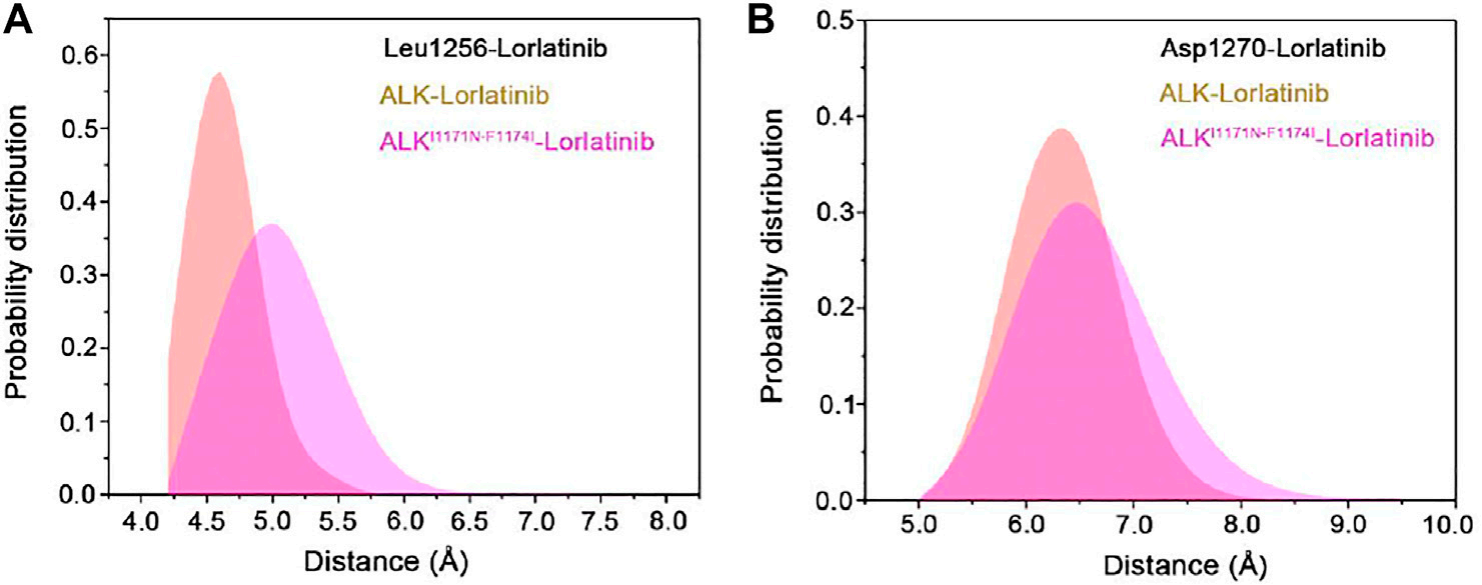
The probability distributions of the two distances (Å) between the centroid of the phenyl moiety of the lorlatinib and the C*α* atoms of Leu1156 **(A)** and Asp1270 **(B)** in the wild-type and the double mutant systems.

The binding free energy calculations, residue decomposition of the total binding free energy, and hydrogen bond analysis in the four systems revealed the decreased binding affinity in the lorlatinib-bound ALK upon the double mutations and highlighted the different residue contributions. In order to further reveal the binding modes of different inhibitor-bound ALK complexes, the representative structural complexes were extracted for each system using cluster analysis (Shao et al., 2007). As shown in Figure 8A, in the ATP-binding site of wild-type and double mutant I1171N/F1174I ALK, the critical residues containing the gatekeeper residue Leu1196, the hinge residues Glu1197 and Met1199, and the Leu1256 at the base of ATP-binding site were significantly disturbed in response to the double mutations. These disturbances caused the upward movement of the lorlatinib in the ATP-binding site of the double mutant, losing the key hydrogen bonding interactions between the hinge residues Glu1197, Met1199 and the lorlatinib, which were in line with the hydrogen bond analysis (Figure 7). Besides, in the mutated site (Figure 8B), the double mutations I1174N/F1174I had a marked effect on the conformational changes of the D^1270^F^1271^G motif and the gatekeeper residue Leu1196. To further reveal the impact of double mutations on the upward movement of lorlatinib in the ATP-binding site, the probability distributions of the two distances between the centroid of the phenyl moiety of the lorlatinib and the C*α* atoms of Leu1156 and Asp1270 were analyzed for all the MD snapshots. As shown in Figure 9A, the peak distance between the C*α* atoms of Leu1156 and the centroid of the phenyl moiety of the lorlatinib was ∼4.5 Å in the wild-type system, while in the double mutant it increased at ∼5.0 Å. Also, as shown in Figure 9B, although the peak distance the C*α* atoms of Asp1270 and the centroid of the phenyl moiety of the lorlatinib was similar (∼6.5 Å) in both systems, the distance distribution was more flexible in range of 7.0–8.0 Å in the double mutant compared to the wild-type system. Together, these data strengthened the argument that the double mutations generated the upward movement of lorlatinib in the ATP-binding site of ALK.

**FIGURE 10:**
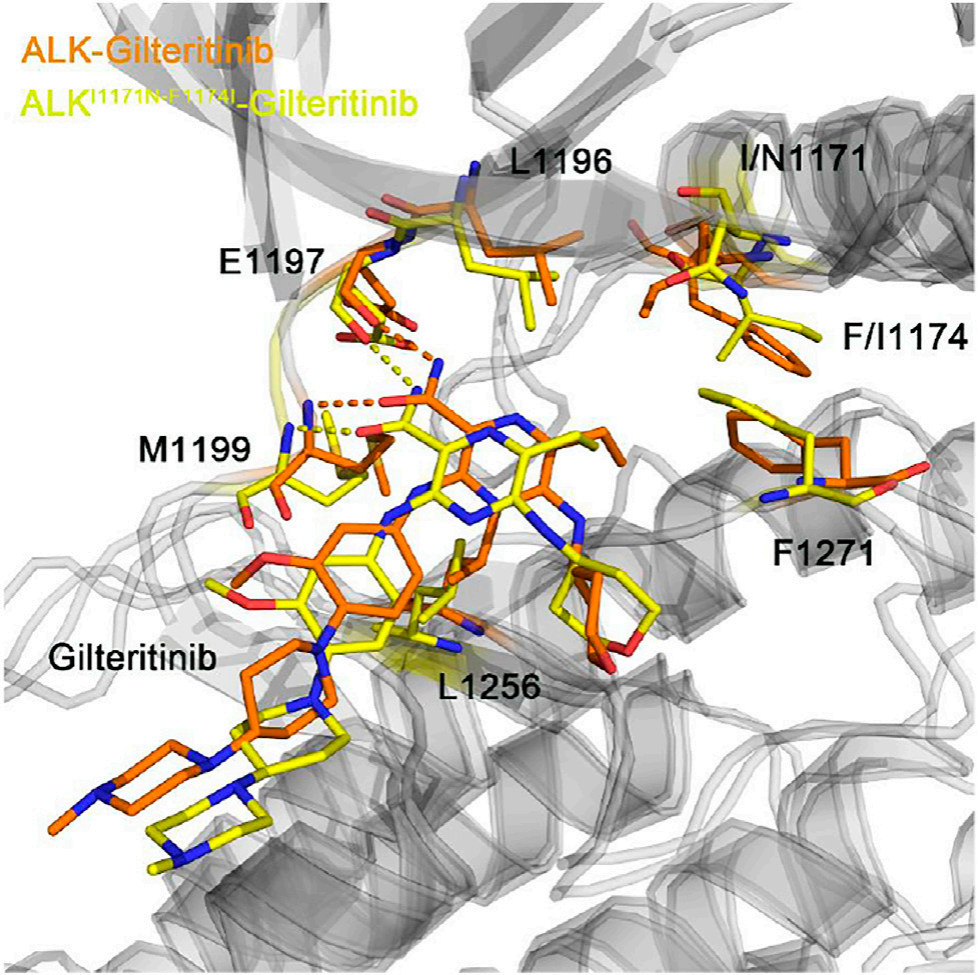
The backbone superimposition of the representative conformation of double mutant I1171N/F1174I gilteritinib-bound ALK to the wild-type structural complex.

Figure 10 showed the representative structural complexes of ALK–gilteritinib in both wild-type and double mutant systems. In the ATP-binding site, the conformations of the critical residues L1196, Glu1197, Met1199, and Leu1256 were similar in both wild-type and double mutant systems. Importantly, both hydrogen bonds were formed between the amide moiety of the gilteritinib and the hinge backbone of Glu1197 and Met1199 in the wild-type and double mutant systems, which were in good agreement with the hydrogen bond analysis (Figure 7). In addition, in the mutated site, the conformations of the DFG motif and the gatekeeper Leu1196 were not markedly disturbed upon the double mutations. As a result, the minor effect of the double mutations on the critical residues of the ATP-binding site and the DFG motif rendered the gilteritinib remaining bound to the double mutant I1171N/F1174I ALK.

## Conclusion

In the present study, multiple replicas of MD simulations, MM-GBSA binding free energy calculations, and coupled domain analysis were performed to decipher the mechanism of how gilteritinib overcomes lorlatinib-resistance in the double mutant ALK I1171N/F1174I. The binding affinities of the lorlatinib and gilteritinib to both the wild-type and double mutant ALK could be generally predicted by virtue of the binding free energies using MM-GBSA calculations. The energy decomposition analysis indicated the difference of the electrostatic and van der Waals interactions were contributed by the conserved residues in the ATP-binding site. Specially, the hinge residues Leu1197 and Met1199 as well as the residue Leu1256 at the base of ATP-binding site had a significant influence on the binding affinities of lorlatinib to the wild-type and double mutant, which were reflected by the hydrogen bonding and hydrophobic interactions. Moreover, the structural analysis revealed that the double mutations I1171N/F1174I yielded the upward movement of the lorlatinib in the ATP-binding site, supporting the predictions on the binding affinities through the MM-GBSA free energy calculations. We anticipate that this study can help to uncover a deeper insight into the mechanism of gilteritinib overcoming lorlatinib-resistance in the double mutant ALK I1171N/F1174I and offer useful information for the design of novel ALK inhibitors.

## Data Availability

The original contributions presented in the study are included in the article/Supplementary Material, further inquiries can be directed to the corresponding authors.
