# Supplementary material for: Deciphering the Mechanism of Gilteritinib Overcoming Lorlatinib Resistance to the Double Mutant I1171N/F1174I in Anaplastic Lymphoma Kinase
Source: Front Cell Dev Biol. 2021 Dec 23;9:808864. doi: 10.3389/fcell.2021.808864 (PMC8733690; doi:10.3389/fcell.2021.808864)
Supplement: Supplementary file 1 [file DataSheet1.pdf]

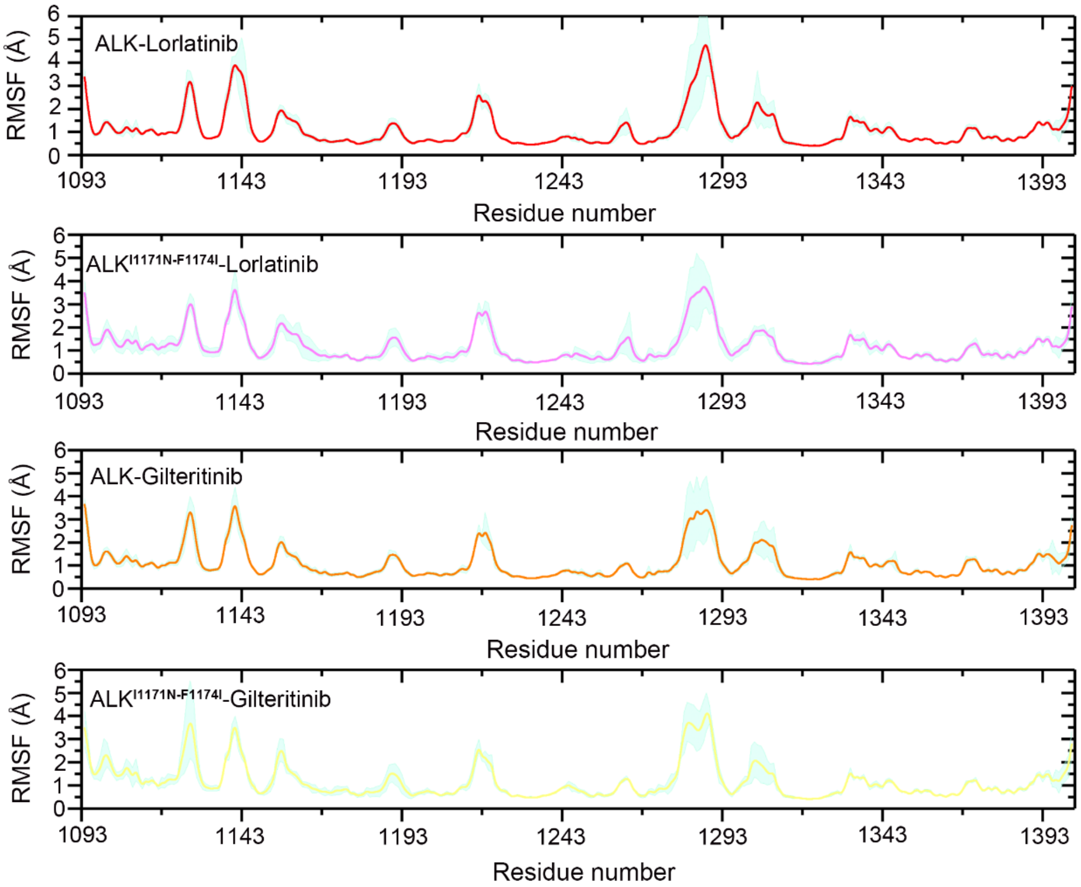


**Figure S1:** The root-mean-square fluctuation (RMSF) profiles of the C$\alpha$ atoms of ALK calculated using all frames of the 15 independent MD simulations for each state. The SEM error bars show RMSF for 15 replicas with respect to the average value.

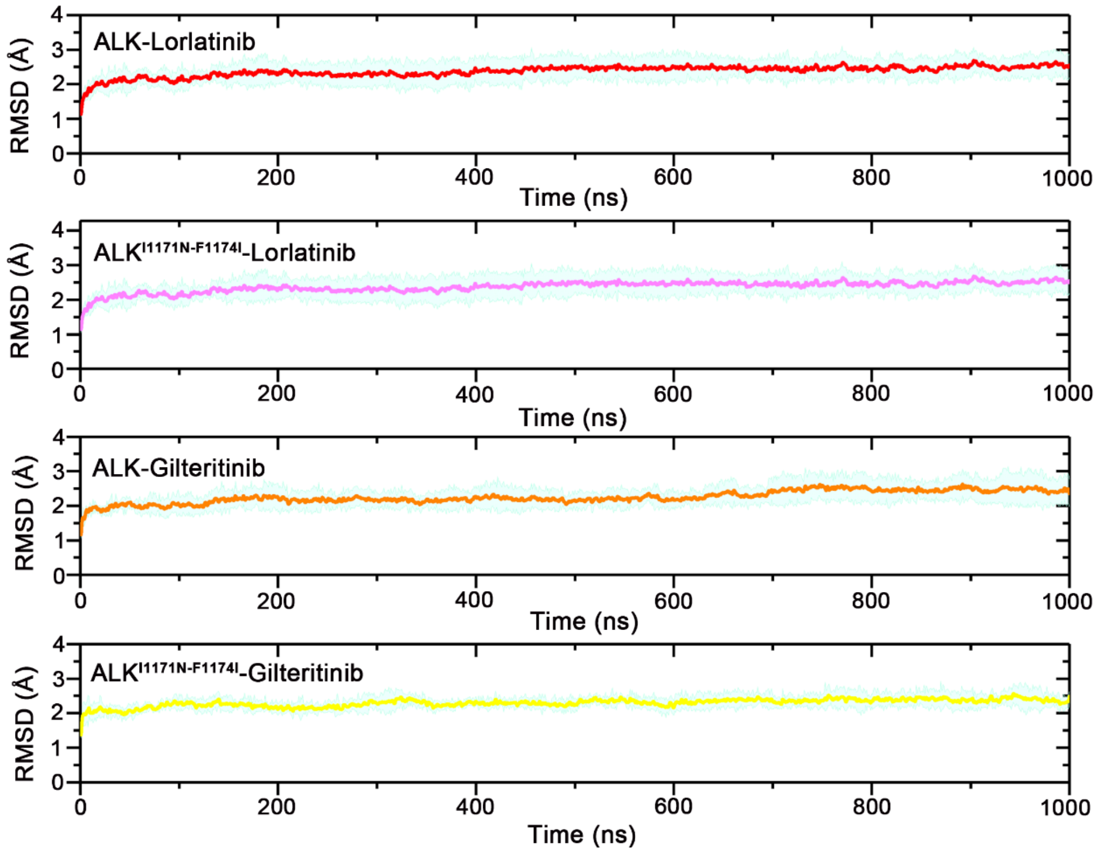


**Figure S2:** The root-mean-square deviation (RMSD) profiles, calculated using the C$\alpha$ atoms of ALK based on all frames of the 15 independent MD simulations for each state. The SEM error bars show RMSF for 15 replicas with respect to the average value.

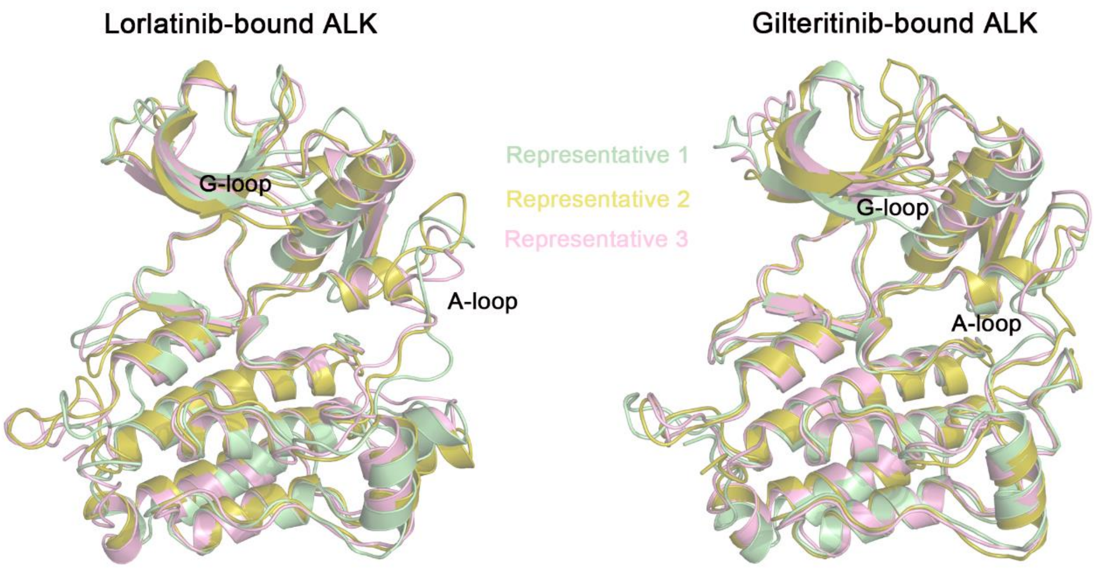


**Figure S3:** Backbone superimposition of the most representative conformation from the clusters C1 (PC1: 0~10, PC2: 0~10) (palegreen), C2 (PC1: -14~-6, PC2: -6~5) (olive), and C3 (PC1: -23~-18, PC2: -5~5) (pink) for the wild-type lorlatinib-bound ALK as well as the clusters C1 (PC1: -17~10, PC2: -10~0) (palegreen), C2 (PC1: -11~-5, PC2: 10~19) (olive), and C3 (PC1: 10~25, PC2: -5~0) (pink) for the wild-type gilteritinib-bound ALK.

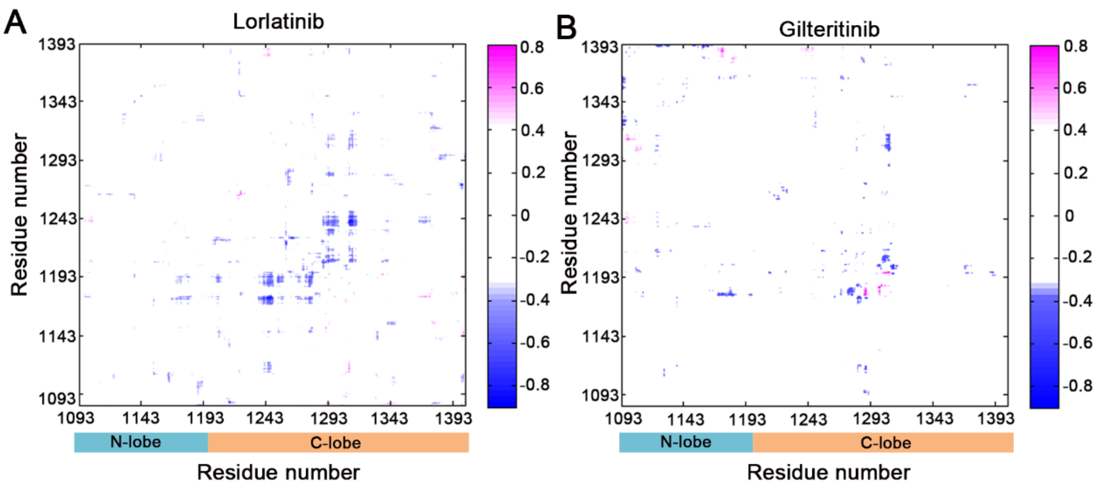


**Figure S4:** The difference of the cross-correlation ($CC_{ij}$) matrix between the ALK–lorlatinib and ALK$^{I1171N\text{-}F1174I}$–lorlatinib (A), and between the ALK–gilteritinib and ALK$^{I1171N\text{-}F1174I}$–gilteritinib (B). The correlated motions are colored by red ($CC_{ij} > 0$), while the anti-correlated motions are colored by blue ($CC_{ij} < 0$). Color scales are shown at the right.
